# Decreased Serum Levels of Mature Brain-Derived Neurotrophic Factor (BDNF), but Not Its Precursor proBDNF, in Patients with Major Depressive Disorder

**DOI:** 10.1371/journal.pone.0042676

**Published:** 2012-08-03

**Authors:** Taisuke Yoshida, Masatomo Ishikawa, Tomihisa Niitsu, Michiko Nakazato, Hiroyuki Watanabe, Tetsuya Shiraishi, Akihiro Shiina, Tasuku Hashimoto, Nobuhisa Kanahara, Tadashi Hasegawa, Masayo Enohara, Atsushi Kimura, Masaomi Iyo, Kenji Hashimoto

**Affiliations:** 1 Division of Clinical Neuroscience, Chiba University Center for Forensic Mental Health, Chiba, Japan; 2 Department of Psychiatry, Chiba University Graduate School of Medicine, Chiba, Japan; 3 Research Center for Child Mental Development, Chiba University Graduate School of Medicine, Chiba, Japan; Rikagaku Kenkyūsho Brain Science Institute, Japan

## Abstract

**Background:**

Meta-analyses have identified serum levels of brain-derived neurotrophic factor (BDNF) as a potential biomarker for major depressive disorder (MDD). However, at the time, commercially available human ELISA kits are unable to distinguish between proBDNF (precursor of BDNF) and mature BDNF because of limited BDNF antibody specificity. In this study, we examined whether serum levels of proBDNF, mature BDNF, and matrix metalloproteinase-9 (MMP-9), which converts proBDNF to mature BDNF, are altered in patients with MDD.

**Methodology/Principal Findings:**

Sixty-nine patients with MDD and 78 age- and gender-matched healthy subjects were enrolled. Patients were evaluated using 17 items on the Structured Interview Guide for the Hamilton Depression Rating Scale. Cognitive impairment was evaluated using the CogState battery. Serum levels of proBDNF, mature BDNF, and MMP-9 were measured using ELISA kits. Serum levels of mature BDNF in patients with MDD were significantly lower than those of normal controls. In contrast, there was no difference in the serum levels of proBDNF and MMP-9 between patients and normal controls. While neither proBDNF nor mature BDNF serum levels was associated with clinical variables, there were significant correlations between MMP-9 serum levels and the severity of depression, quality of life scores, and social function scores in patients.

**Conclusions/Significance:**

These findings suggest that mature BDNF may serve as a biomarker for MDD, and that MMP-9 may play a role in the pathophysiology of MDD. Further studies using larger sample sizes will be needed to investigate these results.

## Introduction

Accumulating evidence suggests that brain-derived neurotrophic factor (BDNF) plays a key role in the pathophysiology of major depressive disorder (MDD), as well as the therapeutic mechanisms of antidepressants [1]–[6]. Previously, we reported that BDNF serum levels in patients with MDD were significantly lower than those of healthy controls, and that there was a negative correlation between BDNF serum levels and the severity of depression in patients [7]. Furthermore, decreased serum levels of BDNF in antidepressant naïve patients with MDD, recovered to levels associated with amelioration of depressive symptoms, after antidepressant treatment [7]. Three meta-analyses [8]–[10] and a study using a large sample size [11] confirmed our previous findings. Therefore, it is likely that the accurate measurement of blood BDNF levels could serve as a potential biomarker for MDD [6].

Mature BDNF is initially synthesized as a precursor protein, preproBDNF, in the endoplasmic reticulum. Following cleavage of the signal peptide, proBDNF is converted to mature BDNF by extracellular proteases, such as matrix metalloproteinase-9 (MMP -9) and plasmin [6], [12]–[16]. It was initially thought that only secreted mature BDNF was biologically active, and that proBDNF, which localizes intracellularly, served as an inactive precursor. However, new evidence shows that proBDNF and mature BDNF elicit opposing effects via the p75^NTR^ and TrkB receptors, respectively, and that both proBDNF and mature BDNF play important roles in several physiological functions [6], [12]–[14]. Considering the important roles of both proBDNF and mature BDNF in physiological functions, it would be informative to measure individual levels of proBDNF and mature BDNF in the body fluids of human subjects [6], [17]. Although BDNF levels in human blood can be measured using commercially available human BDNF ELISA kits, due to the limited specificity of the BDNF antibody, early versions of these kits were unable to distinguish between proBDNF and mature BDNF [18]. Very recently, we reported that serum levels of proBDNF and mature BDNF in healthy subjects were measurable using newly available human proBDNF and BDNF ELISA kits [18].

This study aimed to determine whether serum levels of proBDNF and mature BDNF were altered in patients with MDD. We also investigated MMP-9 serum levels, as MMP-9 plays a role in the conversion of proBDNF to mature BDNF [15], [16]. Since it is also known that patients with MDD suffer cognitive impairment [19]–[21] even in remission [22], we examined the correlation between serum levels of proBDNF, mature BDNF, and MMP-9, with clinical variables, including depression and cognition, in patients with MDD.

## Materials and Methods

### Participants

Sixty-nine patients with MDD and 78 age-matched healthy controls were enrolled (**Table 1**). All patients were outpatients and met DSM-IV criteria for MDD [23]. There were no specific medication criteria for inclusion. Sixty-five patients were treated with antidepressants. Two of the four patients who were antidepressant therapy naïve, had been treated with anxiolytics. Control subjects were screened using the Structured Clinical Interview for DSM-IV Axis I Disorders, Non-Patients Edition, and were required to not have an Axis I disorder, according to DSM-IV criteria [23]. Study investigators made a concerted effort to recruit healthy controls who matched patients on age, male/female ratio, education, premorbid intelligence quotient (IQ) (as assessed by the Japanese Adult Reading Scale-25 version, which is the Japanese version of the National Adult Reading Test), body mass index, and smoking status. Smoking status was dichotomized into current smokers versus non-smokers. Exclusion criteria for subjects in both groups included any current or past history of neurological disorders, including head injury, cerebral vascular disorders, epilepsy, alcohol or drug abuse. Subjects who rarely used personal computers were excluded from the study. Prior to commencement of the study, all subjects provided written informed consent, after receiving a full explanation of the study as well as any potential risks and benefits of study participation. Our samples in the current study consist of all such patients with MDD who are not severe depressive state and possess the ability to agree to join the research. The study was approved by the Ethics Committee of Chiba University Graduate School of Medicine (Chiba, Japan) and performed in accordance with the Declaration of Helsinki II.

**Table 1:** Demographic data of subjects.

Characteristics	Patients (n = 69)	Controls (n = 78)	P values
Gender (male/female)	32/37	32/46	0.514
Age (year olds)	40.5±9.7 (20–60)	37.2±9.8 (20–59)	0.098
Smoking status (current/non-smoker)	20/49	19/59	0.526
Premorbid IQ	105.6±9.4 (85–120)	104.0±8.2 (87–118)	0.308
Education (years)	13.8±2.2 (9–18)	14.0±2.0 (12–18)	0.589
Body mass index	22.7±4.3 (15.0–35.9)	22.0±3.3 (17.1–34.3)	0.156
WHOQOL-BREF score	2.63±0.54 (1.27–3.69)	3.78±0.38 (1.96–2.88)	<0.001*
SASS score	26.5±8.3 (8–44)	41.7±5.4 (29–56)	<0.001*
CogState composite score	−0.46±0.80 (−2.85–1.02)	0.00±0.352 (−0.85–0.83)	<0.001^+^
Age of first depressive episode	33.0±10.0 (11–55)		
Duration of illness (years)	7.2±7.3 (0–29)		
Duration of untreated illness (years)	1.0±1.7 (0–9)		
SIGH-D score	11.8±5.5 (0–24)		

Data show the mean ± SD. Figures in parenthesis represent the range.

WHOQOL-BREF: World Health Organization Quality of Life-Short Version.

SASS: Social Adaptation Self-evaluation Scale, SIGH-D: 17 items of the Structured Interview Guide for the Hamilton Depression Rating Scale.

* Student's t-test, ^+^Mann-Whitney's U-test.

### Assessment of clinical variables

Depression was assessed using 17 items of the Structured Interview Guide for the Hamilton Depression Rating Scale (SIGH-D) [24]. Quality of life (QOL) was assessed using WHOQOL-BREF. Social function was assessed using the Japanese version of the Social Adaptation Self-evaluation Scale (SASS) [25], which is a validated self-evaluation scale for assessment of social functioning [26].

Cognitive impairment was assessed using the Japanese language version of the CogState battery, a rapid, automatically administered computerized battery which assesses verbal learning, visual learning, speed of processing, attention/vigilance, visual working memory, spatial working memory, reasoning and problem solving, and social cognition [27]. The primary measure from each task of the CogState battery was standardized by creating Z-scores. The healthy control mean was set to zero and the standard deviation set to one, following the methodological procedure used by Keefe et al. [28]. A composite score was calculated by averaging all Z-scores from the eight primary measures of the CogState battery. The composite score of the CogState battery correlated well with the composite score of the Brief Assessment of Cognition in Schizophrenia (BACS), Japanese-language version [27] and the composite score of the MATRICS Consensus Cognitive Battery [29]. Therefore, we used these composite scores as representative values for cognition.

### Measurement of proBDNF, mature BDNF, and MMP-9 serum levels

Serum samples from control subjects were collected between 9:00 and 15:00, and stored at −80°C until use. Serum levels of proBDNF, mature BDNF, and MMP-9 were measured using the human proBDNF ELISA Kit (Adipo Bioscience, Santa Clara, CA, USA), the human BDNF ELISA Kit (Adipo Bioscience, Santa Clara, CA, USA), and the human MMP-9 ELISA Kit (R&D Systems, Minneapolis, MN, USA), respectively. To minimize assay variance, serum levels of proBDNF, mature BDNF, and MMP-9 from each subject were measured on the same day. All experiments were performed in duplicate. Protocols were performed according to the manufacturer's instructions. The optical density of each well was measured using an automated microplate reader (Emax; Molecular Devices, Sunnyvale, CA, USA).

### Statistical analyses

The data show the mean ± standard deviation (SD). Chi-squared test was used for categorical variables. Parametric Student's *t*-test or non-parametric Mann-Whitney test was used to compare two groups. Correlations with clinical variables were performed using Pearson's correlation or Spearman's correlation. A *p* value of <0.05 was judged as statistically significant. All analyses were carried out using SPSS version 19.0 (SPSS Inc, Chicago, IL, USA).

## Results

### Demographic data and clinical variables

The demographic information and clinical variables of subjects are presented in **Table 1**. Age, gender, estimated premorbid IQ, education, body mass index, and smoking status did not differ between the two groups. The WHOQOL-BREF and SASS scores in patients with MDD were significantly lower than those of controls, indicating that patients with MDD had a decreased QOL and poor social function. Furthermore, the composite scores of the CogState battery in patients with MDD were significantly lower than those of healthy controls, indicating cognitive impairment in this patients group (**Table 1**).

### Serum levels of proBDNF, mature BDNF, and MMP-9

**Figure 1:**
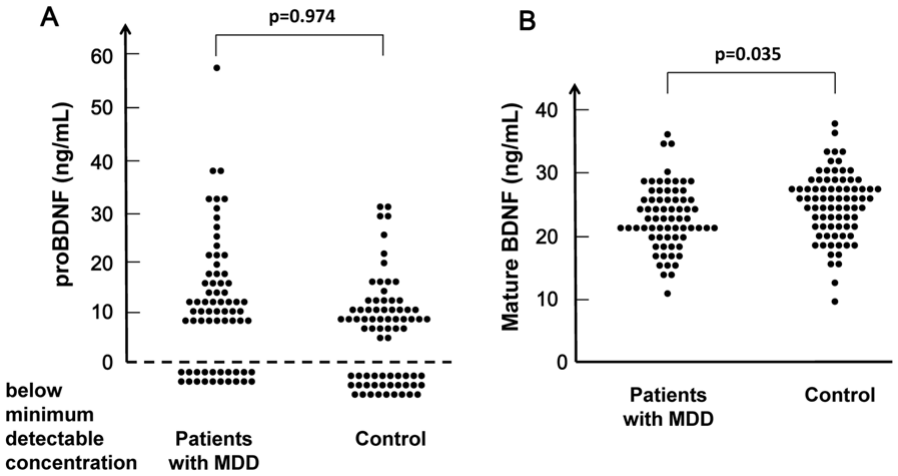
Scatter plot of proBDNF and mature BDNF serum levels in patients with MDD and healthy controls. (A): Serum levels of proBDNF in 20 patients with MDD and 29 healthy subjects were below the minimum detectable concentration (0.5 ng/mL) of the proBDNF ELISA kits. Serum levels of proBDNF in patients with MDD did not differ from those of normal controls. (B): In contrast, serum levels (21.09±5.60 ng/mL) of mature BDNF in patients with MDD, were significantly lower than those (23.11±5.90 ng/mL) of normal controls.

In 20 patients and 29 controls, serum levels of proBDNF were below the minimum detectable concentration (0.5 ng/mL) of the proBDNF ELISA kits. Chi-squared testing showed no difference (*x* ^2^ = 1.106, p = 0.293) between these groups. Serum levels of proBDNF (10.68±11.28 ng/mL) in patients (n = 49) were not different (p = 0.974) from those (8.90±7.98 ng/mL) in controls (n = 49) (**Figure 1A**).

In contrast, serum levels of mature BDNF (21.09±5.60 ng/mL) in patients (n = 69) were significantly (p = 0.035) lower than those (23.11±5.90 ng/mL) of controls (n = 78) (**Figure 1B**).

**Figure 2:**
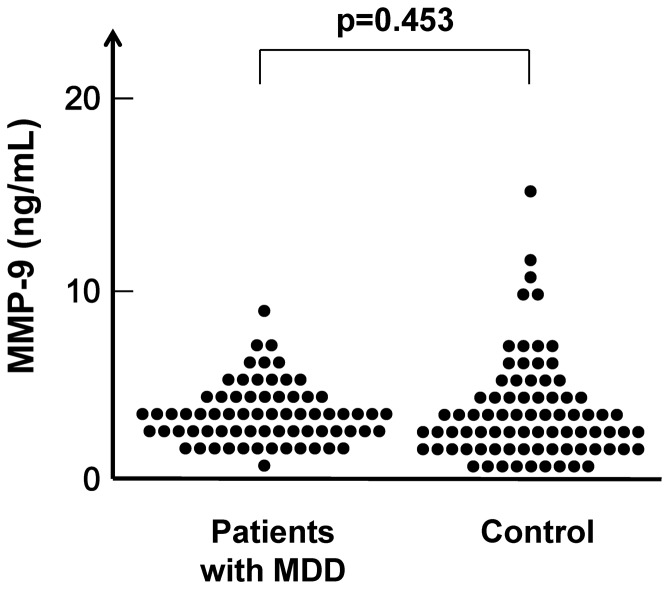
Scatter plot of MMP-9 serum levels in patients with MDD and healthy controls. Serum levels of MMP-9 in patients with MDD did not differ from those of normal controls.

Serum levels of MMP-9 (4.52±1.69 ng/mL) in patients (n = 69) were no different (p = 0.453) from those (4.63±2.69 ng/mL) in controls (n = 78) (**Figure 2**).

### Correlations with clinical variables

In subjects with measurable serum levels of proBDNF, there was no correlation between this molecule and clinical variables, such as smoking status, body mass index, duration of illness, duration of untreated illness, SIGH-D scores, WHOQOL-BREF scores, SASS scores, and composite scores of the CogState battery.

There were significant weak correlations between serum levels of mature BDNF and duration of illness (ρ = 0.282, p = 0.019) and body mass index (r = 0.345, p = 0.005) in patients (n = 69). However, there was no correlation between mature BDNF levels, and other clinical variables, such as smoking status, duration of untreated illness, SIGH-D scores, WHOQOL-BREF scores, SASS scores, and composite scores of the CogState battery.

**Figure 3:**
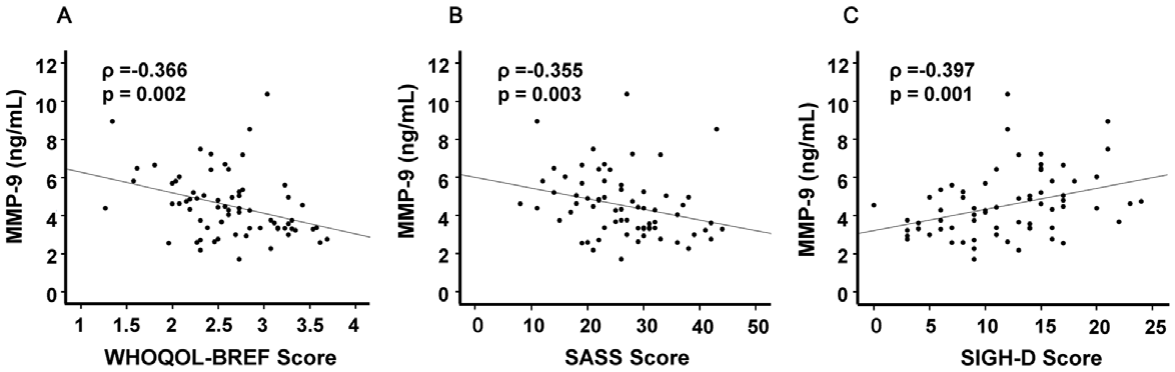
Relationships between MMP-9 serum levels and clinical variables in patients with MDD. (A): There was a significant negative correlation (ρ = −0.366, p = 0.002) between MMP-9 serum levels and WHOQOL-BREF scores. (B): There was a significant negative correlation (ρ = −0.355, p = 0.003) between MMP-9 serum levels and SASS scores. (C): There was a significant positive correlation (ρ = 0.397, p = 0.001) between MMP-9 serum levels and the SIGH-D score.

Interestingly, there were significant correlations between MMP-9 levels and WHOQOL-BREF scores (ρ = −0.366, p = 0.002) (**Figure 3A**), SASS scores (ρ = −0.355, p = 0.003) (**Figure 3B**), and the SIGH-D score (ρ = 0.397, p = 0.001) (**Figure 3C**) in patients (n = 69). In contrast, there was no correlation between MMP-9 levels and other clinical variables, including composite scores of the CogState battery in patients (n = 69) and all subjects (n = 147).

**Figure 4:**
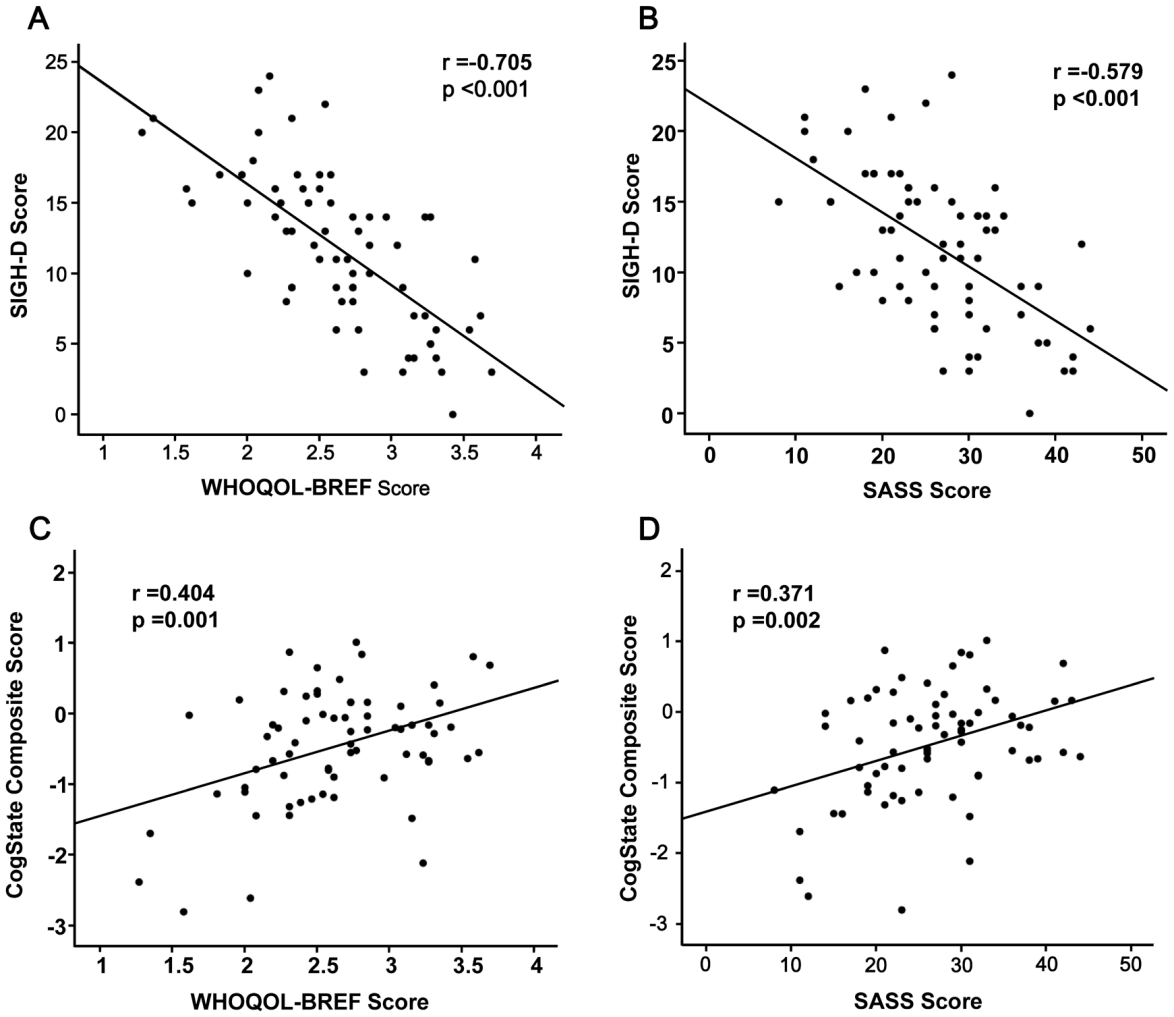
Relationships between clinical variables in patients with MDD. (A): There was a significant negative correlation (r = −0.705, p<0.001) between the SIGH-D score and WHOQOL-BREF score in patients. (B): There was a significant negative correlation (r = −0.579, p<0.001) between the SIGH-D and SASS scores in patients. (C): There was a positive correlation (r = 0.404, p = 0.001) between the CogState composite score and QOL score in patients. (D): There was a positive correlation (r = 0.371, p = 0.002) between the CogState composite score and SASS score in patients.

Next, we examined the relationships between clinical variables in all subjects and patients. There was a high positive correlation (ρ = 0.828, p<0.001) between SASS and WHOQOL-BREF scores in all subjects (n = 147), suggesting a close relationship between QOL and social function. There were negative correlations between SIGH-D and WHOQOL-BREF scores (r = −0.705, p<0.001) and SIGH-D and SASS scores (r = −0.579, p<0.001) in patients (n = 69) (**Figure 4A and 4B**). Moreover, there were positive correlations between CogState composite scores and WHOQOL-BREF scores (r = 0.404, p = 0.001) and SASS scores (r = 0.371, p = 0.002) in patients (n = 69) (**Figure 4C and 4D**). These findings suggest that the symptoms of depression and cognition impinge on the QOL and social function of patients with MDD.

## Discussion

In this study, we found that for patients with MDD, serum levels of mature BDNF, but not proBDNF were significantly lower than those of age- and gender-matched healthy controls. To the best of our knowledge, this is the first report showing decreased serum levels of mature BDNF in patients with MDD. Three meta-analyses and a large cohort study [8]–[11] demonstrated that serum levels of BDNF were significantly lower in patients with MDD, although at the time, the commercially available human BDNF ELISA kits were unable to distinguish between proBDNF and mature BDNF because of the limited specificity of the BDNF antibody [18]. It means therefore, that these studies reported on combined levels of proBDNF and mature BDNF, and not the sole levels of mature BDNF [18]. In contrast, the proBDNF and mature BDNF ELISA kits used in the present study are able to distinguish between pro and mature forms of BDNF in human serum [18]. Therefore, it is likely that a reduction in mature BDNF contributes to the decreased levels of BDNF in patients with MDD, observed in earlier reports [8]–[11].

Previously, we reported that serum levels of BDNF in antidepressant-naïve patients with MDD were significantly lower than those of medicated patients and healthy controls, and that serum levels of BDNF were correlated negatively with the severity of depression [7]. In addition, BDNF serum levels in antidepressant-naïve patients increased after antidepressant treatment [7]. These results were supported by subsequent meta-analyses [8]–[11]. Taken together these findings imply that serum BDNF levels may function as a state biomarker for MDD. However, in this study, we found no correlation between mature BDNF levels and the severity of depression in patients with MDD. Patients enrolled in this study had moderate, but not severe symptoms of depression (SIGH-D score; 0–24) compared with our previous report [7]. It is likely that the lack of correlation between mature BDNF levels and depression severity seen in our study may be due to the absence of severe disease patients within our cohort. A further study using patients with severe depression will be needed to resolve this issue.

In this study, we were unable to measure serum levels of proBDNF in 49 subjects (20 patients and 29 healthy controls), because their values fell below the minimum detectable threshold of the ELISA kit. The manufacturer's instructions state that the minimum detectable concentrations of the proBDNF and mature BDNF ELISA kits are 0.5 ng/mL and 5–8 pg/mL, respectively, indicating that the sensitivity of the proBDNF kit is markedly lower than that of the mature BDNF kit [18]. As mentioned above, proBDNF and mature BDNF have opposing biological functions in the brain and peripheral organs [6], [12]–[14]. Therefore, accurate measurement of serum proBDNF levels in human samples requires the development of a higher sensitivity ELISA kit than is currently available.

MMP-9 plays a key role in synaptic plasticity of the brain, and acts by converting proBDNF to mature BDNF, which in turn results in TrkB activation [15], [16]. A recent study using MMP-9 knock-out mice demonstrated that MMP-9 plays a role in the development of pentylenetetrazole-induced kindling, by converting proBDNF to mature BDNF in the hippocampus [30]. This study, found no difference in serum MMP-9 levels between patients with MDD and healthy controls, consistent with a previous report that detected no changes from the norm in gingival crevicular fluid MMP-9 levels from female patients with depression [31]. Interestingly, we found a positive correlation between serum MMP-9 levels and the severity of depression in patients with MDD, although the role of MMP-9 in the pathophysiology of MDD is currently unknown. One possibility is that MMP-9 expression is increased as a compensatory response to decreases in mature BDNF, in patients with MDD. A recent proteomic approach using plasma samples from a large case-control cohort, demonstrated that plasma levels of MMP-9 in patients with MDD (n = 245) were significantly higher than those of controls (n = 254) [32], a finding that is inconsistent with our data. The reasons underlying this discrepancy are currently unknown. It has also been reported that the *MMP-9* gene polymorphisms are associated with cardiovascular disease and neuropsychiatric disorders, including schizophrenia, and bipolar disorder [33], suggesting that this enzyme may be a pathological mediator in these diseases. Given the opposing functions of proBDNF and mature BDNF, it would be of great interest to study the precise mechanisms controlling the cleavage of proBDNF to mature BDNF [6]. Therefore, further detailed studies will be also necessary to examine the role of MMP-9 in the pathophysiology of MDD.

Finally, there are some limitations to this study that need to be mentioned. The main limitation was the very small cohort size of the medication-naïve patients with severe symptoms of depression. Antidepressant medication is known to increase serum levels of BDNF in patients with MDD [6], [8]–[11]. Therefore, further studies using a larger sample size of medication-naïve patients will be needed. Another limitation was the sensitivity of the human proBDNF ELISA kits, a sensitivity which is significantly lower than that of the human mature BDNF kit [18]. Given the key role of proBDNF/mature BDNF/TrkB signaling in the pathophysiology of MDD [7], [12]–[14], it is imperative to develop proBDNF ELISA kits of greater sensitivity. It would also be of great interest to study the relationships between serum levels of proBDNF and mature BDNF along with levels of extracellular peptidases (e.g., MMP-9, plasmin) that convert proBDNF to mature BDNF.

In conclusion, we found that in patients with MDD, serum levels of mature BDNF, but not proBDNF, were significantly lower than those of healthy controls. Furthermore, we found correlations between serum MMP-9 levels and depression, QOL, and social function in patients, although there were no differences in serum MMP-9 levels between patients and healthy controls. Further studies measuring the serum levels of proBDNF, mature BDNF, and MMP-9 using larger cohorts, particularly cohorts of antidepressant-naïve patients, will be needed.
