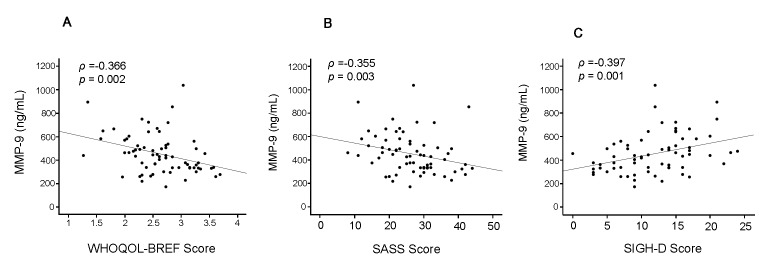# Correction: Decreased Serum Levels of Mature Brain-Derived Neurotrophic Factor (BDNF), but Not Its Precursor proBDNF, in Patients with Major Depressive Disorder

**DOI:** 10.1371/annotation/85a3fa48-980b-4f95-bb43-b33b1c3e0ac6

**Published:** 2013-02-25

**Authors:** Taisuke Yoshida, Masatomo Ishikawa, Tomihisa Niitsu, Michiko Nakazato, Hiroyuki Watanabe, Tetsuya Shiraishi, Akihiro Shiina, Tasuku Hashimoto, Nobuhisa Kanahara, Tadashi Hasegawa, Masayo Enohara, Atsushi Kimura, Masaomi Iyo, Kenji Hashimoto

The authors found that the unit of serum MMP-9 levels was not correct. Therefore, the authors corrected the unit of serum MMP-9 concentration in the Figure 2 and Figure 3. In addition, the authors corrected the following sentence in the Results section:

Serum levels of MMP-9 (452.0 ± 169.0 ng/mL) in patients (n=69) were no different (p=0.453) from those (462.9 ± 269.4 ng/mL) in controls (n=78)(Figure 2).

The corrected Figure 2 can be viewed here: 

**Figure:**
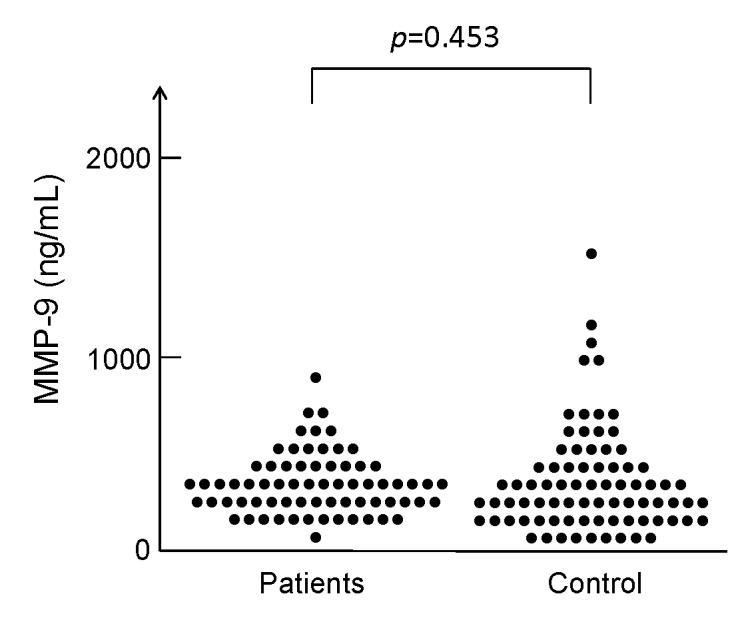


The corrected Figure 3 can be viewed here: 

**Figure:**